# Using Web-Based Social Media to Recruit Heavy-Drinking Young Adults for Sleep Intervention: Prospective Observational Study

**DOI:** 10.2196/17449

**Published:** 2020-08-11

**Authors:** Garrett I Ash, David S Robledo, Momoko Ishii, Brian Pittman, Kelly S DeMartini, Stephanie S O'Malley, Nancy S Redeker, Lisa M Fucito

**Affiliations:** 1 Department of Psychiatry Yale School of Medicine New Haven, CT United States; 2 Pain, Research, Informatics, Medical Comorbidities and Education Center (PRIME) Veterans Affairs Connecticut Healthcare System West Haven, CT United States; 3 Yale School of Public Health New Haven, CT United States; 4 Yale School of Nursing New Haven, CT United States; 5 Yale Cancer Center New Haven, CT United States; 6 Smilow Cancer Hospital Yale-New Haven Hospital New Haven, CT United States

**Keywords:** substance abuse, social media, alcohol drinking, sleep, mobile phone

## Abstract

**Background:**

Novel alcohol prevention strategies are needed for heavy-drinking young adults. Sleep problems are common among young adults who drink heavily and are a risk factor for developing an alcohol use disorder (AUD). Young adults, interested in the connection between sleep and alcohol, are open to getting help with their sleep. Therefore, sleep interventions may offer an innovative solution. This study evaluates social media advertising for reaching young adults and recruiting them for a new alcohol prevention program focused on sleep.

**Objective:**

This study aims to evaluate the effectiveness and cost of using Facebook, Instagram, and Snapchat advertising to reach young adults who drink heavily for a sleep intervention; characterize responders’ sleep, alcohol use, and related concerns and interests; and identify the most appealing advertising content.

**Methods:**

In study 1, advertisements targeting young adults with sleep concerns, heavy alcohol use, or interest in participating in a sleep program ran over 3 months. Advertisements directed volunteers to a brief web-based survey to determine initial sleep program eligibility and characterize the concerns or interests that attracted them to click the advertisement. In study 2, three advertisements ran simultaneously for 2 days to enable us to compare the effectiveness of specific advertising themes.

**Results:**

In study 1, advertisements generated 13,638 clicks, 909 surveys, and 27 enrolled volunteers in 3 months across the social media platforms. Fees averaged US $0.27 per click, US $3.99 per completed survey, US $11.43 per volunteer meeting initial screening eligibility, and US $106.59 per study enrollee. On average, those who completed the web-based survey were 21.1 (SD 2.3) years of age, and 69.4% (631/909) were female. Most reported sleep concerns (725/909, 79.8%) and an interest in the connection between sleep and alcohol use (547/909, 60.2%), but few had drinking concerns (49/909, 5.4%). About one-third (317/909, 34.9%) were identified as being at risk for developing an AUD based on a validated alcohol screener. Among this subsample, 8.5% (27/317) met the final criteria and were enrolled in the trial. Some volunteers also referred additional volunteers by word of mouth. In study 2, advertisements targeting sleep yielded a higher response rate than advertisements targeting alcohol use (0.91% vs 0.56% click rate, respectively; *P*<.001).

**Conclusions:**

Social media advertisements designed to target young adults with sleep concerns reached those who also drank alcohol heavily, despite few being concerned about their drinking. Moreover, advertisements focused on sleep were more effective than those focused on drinking. Compared with previous studies, cost-effectiveness was moderate for engagement (impressions to clicks), excellent for conversion (clicks to survey completion), and reasonable for enrollment. These data demonstrate the utility of social media advertising focused on sleep to reach young adults who drink heavily and recruit them for intervention.

## Introduction

### Background

Heavy alcohol use remains a problem among young adults. Alcohol use disorder (AUD) onset peaks during young adulthood (ie, 18-25 years) [1]. Compared with older adults, young adults report more frequent and heavier alcohol use that is linked to substantial negative consequences including the risk of accidental injury, the primary cause of death among young adults [2,3]. Current alcohol intervention strategies for young adults have modest effects [4-6], and young adults rarely self-identify for specialized alcohol treatment [7,8]. Thus, more research is needed to identify effective alcohol interventions and novel treatment engagement strategies to reduce this substantial public health burden.

Concurrent to their high rates of alcohol consumption, research suggests that many heavy-drinking young adults have poor sleep. In this age group, greater alcohol consumption and alcohol-related consequences are associated with shorter sleep duration, poorer sleep quality, and more delayed bed/wake times [9,10]. In addition, various sleep problems in adolescence predict earlier AUD onset and greater risk of heavy drinking, alcohol-related consequences, and AUD in young adulthood [11-13]. Furthermore, poor sleep in young adults predicts a greater future risk of alcohol-related problems [14].

Although most heavy-drinking young adults do not seek alcohol treatment [7,8], sleep interventions may be a means to identify those who drink heavily and engage them in treatment. Our previous formative work showed that young adults are interested in the connection between sleep and alcohol and are open to getting help for their sleep [15]. Thus, sleep could be a novel intervention target to engage young adults and promote alcohol behavior change [15,16].

The promise of this approach raises the question of the best vehicle for recruitment. Young adults visit health care providers in person less often [17] but go on the web for health information and use mobile health apps more often as compared to adolescents [18]. Among these mobile health apps, sleep is the third most popular topic, following fitness and nutrition [18]. Moreover, heavy-drinking young adults may prefer mobile sleep interventions over in-person models [15]. Thus, considering young adults’ health practices and preferences, one possible recruitment strategy is to utilize technology.

Previous research suggests that social media is a cost-effective method for recruiting young adults [19-26] as well as hard-to-reach populations and those affected by specific physical or mental health conditions [27-29]. Urban women at high risk of HIV were recruited into an HIV prevention intervention [23], young adult smokers were recruited into a smoking cessation intervention [26], and African American women with elevated blood pressure were recruited into a physical activity and nutrition intervention [28]. The majority of studies evaluated Facebook for this purpose [19,20,23,25-29]. Other social media platforms, such as Instagram and Snapchat, have emerged and are highly popular among young adults [30], but few studies have researched them as recruitment tools [21,22].

This is an important research gap because the platforms differ both practically and theoretically. The most salient practical differences include the following: (1) Instagram and Snapchat are image based with minimal text, whereas Facebook uses both images and text, (2) Snapchat immediately deletes content after viewing, whereas Instagram and Facebook archive it, and (3) Snapchat restricts interaction with users with an existing relationship (ie, have exchanged screen names), whereas Instagram and Facebook allow public interactions. The uses and gratifications theory, which rests on the premise that consumers actively choose media with the intention of fulfilling specific needs, has unveiled some ramifications of these differences among young adults. First, image-based social media (Instagram and Snapchat) led to decreased loneliness and increased happiness and satisfaction with life, whereas Facebook and other text-based media did not [31]. Second, Snapchat led to deeper levels of personal disclosure than other platforms [32-34]. A qualitative analysis suggested this could be due to Snapchat’s immediate deletion of communications assuaging user apprehension about their future ramifications and Snapchat’s restriction of communication to existing relationships facilitating more trusting connections than the communications with strangers allowed on other platforms [32-34]. It is not known whether these differing uses and gratifications between the platforms lead to differing utility for research recruitment.

### Objectives

The goal of this study is to evaluate social media advertising to recruit young adults who report heavy alcohol use for a mobile sleep intervention. The aims are to evaluate the effectiveness and cost of using Facebook, Instagram, and Snapchat advertising to reach young adults with sleep concerns and a subpopulation with heavy drinking behaviors; characterize sleep, alcohol use, and related concerns and interests among responders; and identify advertising content that yielded the highest response rate. These data may suggest new ideas for recruitment or innovative mobile interventions to address poor sleep and heavy drinking among young adults.

## Methods

The methods described below are based on our previous investigation of social media advertising for a different population (heavy-drinking smokers) [35]. Our previous study and this study shared the primary objective of evaluating the effectiveness and cost of using social media advertising to recruit from the population of interest.

### Screening Process Overview

The target population of the web survey was young adult (aged 18-25 years) heavy drinkers at risk for AUD (defined below, Drinking Habits) who were interested in participating in a sleep intervention (trial registration: NCT036589); lived in the greater New Haven, Connecticut area (to complete in-person visits); owned a smartphone (to complete the mobile intervention); and were literate in English. Research staff contacted volunteers who met these preliminary criteria to verify survey-reported drinking patterns and screened out exclusion criteria: confounders of circadian rhythm (night shift work or >2 time zones travel in the past month or next 3 months), severe AUD, or unsafe to complete transdermal ankle alcohol monitoring (eg, peripheral vascular condition). Eligible and interested volunteers were invited to an intake visit at our research lab. Although the survey has been ongoing, we have restricted our analysis to (1) all responses collected for the first 3 months (January 14 to April 18, 2019) to describe the population of responders and the overall campaign effectiveness and (2) responses to specific advertisements over a short trial period (October 5 and 6, 2019) to compare the effectiveness of advertising themes. The study and screening process were approved by the Yale University Institutional Review Board.

### Study 1: Description of the Population of Responders and Overall Campaign Effectiveness

#### Facebook, Instagram, and Snapchat Recruitment

We ran advertisements through Facebook, Instagram, and Snapchat paid advertising services for 41 days over a 3-month period between January 14 and April 18, 2019. Our campaign included 4 advertisements appearing on the Facebook, Instagram (desktop and mobile), and Snapchat (mobile) interfaces of individuals in our age group (18-25 years). Advertisements were turned on, each for a period of days, until we enrolled our target number of volunteers for that study period (n=32). We specified a spending limit of US $25 per day for Facebook and Instagram (combined) and US $50 per day for Snapchat (Snapchat’s minimum).

**Figure 1:**
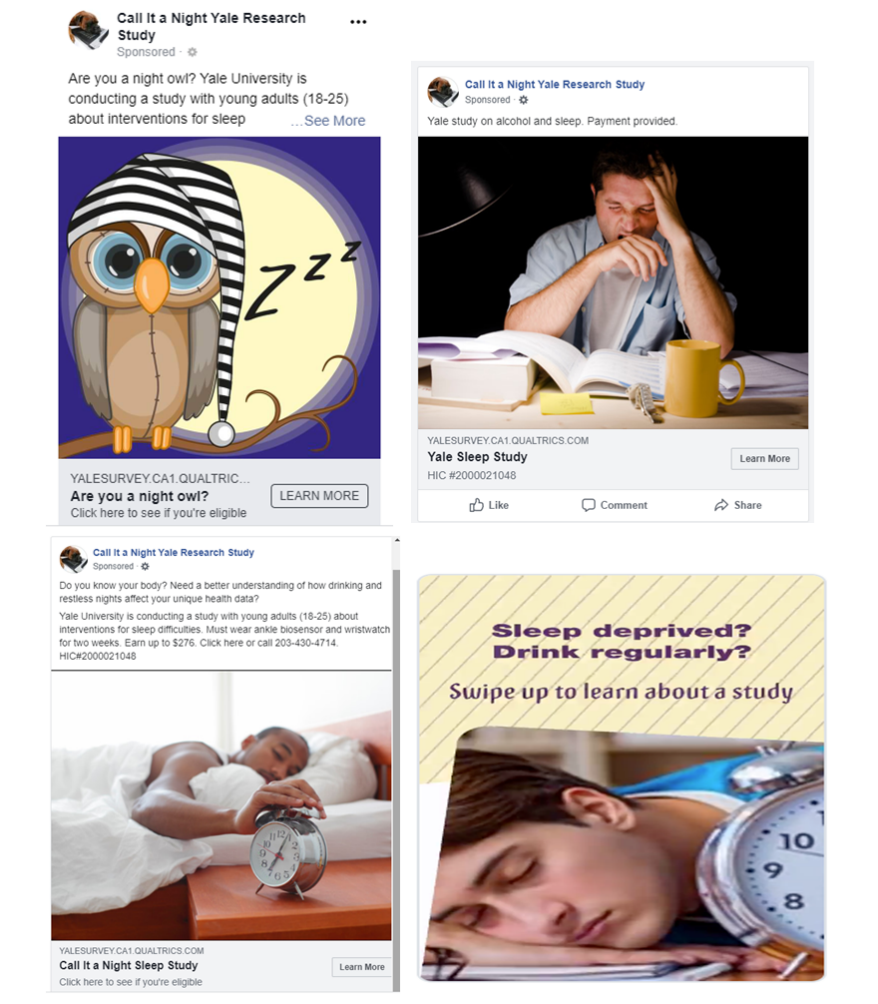
Examples from advertising campaign (study #1) designed to reach young adults with heavy drinking for a sleep intervention.

We restricted the geographic radius to a range that made travel to New Haven feasible without compromising the daily number of times the advertisement was displayed (ie, impressions). The advertising platforms reported a greater number of users within 25 miles of New Haven for Snapchat (383,000) than for Facebook and Instagram combined (220,000), which was counterbalanced by selecting a narrower radius for Snapchat (10 miles) than for Facebook and Instagram (25 miles). The 4 advertisements featured various combinations of the following 3 themes: (1) sleep concern (eg, “sleep deprived?”), (2) drinking behavior (eg, “drink regularly?”) although not drinking concern, and (3) health behavior intersection (eg, “need a better understanding of how drinking and restless nights affect your unique health data?”; Figure 1). Images featuring only men, which have been previously reported to yield higher conversion rates than advertisements featuring women [36]. Wording style was taken from our previous successful social media campaign [16].

Facebook and Instagram run on a shared platform, and Snapchat runs on its own platform. Facebook and Snapchat allocate advertising space using an *auction* process based on the spending *bid* of the advertiser, relevance to the user (ie, web analytic estimated rate of the user acting upon the advertisement), and advertisement quality (ie, past user experience survey results) [37,38]. We used the bid-optimizing algorithms offered by Facebook and Snapchat targeting the lowest cost per click. Facebook’s auctioning and bid optimization include the Instagram space.

Each platform monitored the number of impressions, total reach (ie, number of people seeing the advertisements), advertisement clicks, and total cost for all advertisements. These data allowed us to evaluate efficacy and cost-effectiveness.

#### Survey Procedures

The Yale Institutional Review Board approved this protocol. Study advertisements could be clicked on Facebook and Instagram or *swiped up* on Snapchat. By clicking on Facebook or Instagram advertisements, users were directed to a web-based survey to screen for initial eligibility. By *swiping up* on Snapchat, users were connected to our study website, which provided a description of the study [39] and a link to the same eligibility survey. Snapchat did not allow a direct link to the web-based survey because it found that our privacy policy lacked enough explanation to be directly linked from an advertisement. The eligibility survey was administered by a HIPAA-compliant web interface (Qualtrics). It began with an overview of the study: (1) we are looking for young adults who want to improve their sleep and drink alcohol, (2) we are testing different mobile health strategies for improving sleep, and (3) the procedures include web-based sleep education along with sleep and alcohol biosensors and diaries. It then provided information about study compensation (US $276), funding source, research team contact details, and confidentiality. Volunteers were informed that they had the option to complete screening by phone instead of using the web (none utilized). Before completing the survey, volunteers were asked to affirm that they were older than 18 years of age, understood the information, and wished to proceed. Volunteers were then encouraged to provide their email addresses and phone numbers to be contacted if eligible.

#### Survey Items

The survey included 29 questions that took approximately 5-10 min to complete. “I choose not to answer” was an option for all multiple-choice items.

#### Study Interest and Referral Source

The initial questions asked how volunteers heard about the study and what made them interested in participating. Volunteers could select one or more options among the multiple options (concerned about sleep, concerned about drinking, interest in sleep, interest in alcohol drinking, interest in the connection between sleep and alcohol drinking, and others).

#### Demographics

Volunteers were asked about their age and sex.

#### Basic Exclusion Criteria

Volunteers were asked if they owned a smartphone and whether they could read and write in English.

#### Alcohol Quantity and Frequency

Volunteers were asked about their quantity of drinking (average standard drinks consumed on each day of the week) over the past 30 days [40] and frequency of any heavy drinking (≥5 drinks among men vs ≥4 drinks among women) over the past 14 days. They were advised to report in standard drinks (12 ounces of beer, 5 ounces of wine, and 1.5 ounces of hard liquor shot or mixed drink).

#### AUD Risk Status

Volunteers also completed the consumption questions of the Alcohol Use Disorders Identification Test (AUDIT-C): frequency of any drinking, typical drinks per occasion, and frequency of ≥6 drinks. The AUDIT-C scores of ≥7 for men and ≥5 for women have strongly distinguished at-risk drinking for young adults compared with reference standards (area under receiver operating characteristic curve=0.89) [41]. Volunteers who met the AUDIT-C threshold were deemed preliminarily eligible. Final eligibility was verified by telephone. Volunteers had to report ≥3 episodes of heavy drinking in the past 14 days.

#### Sleep

Volunteers rated their sleep quality over the past 7 days using a single-item Likert-type scale with anchors 1=“very poor” and 5=“very good.” This single item correlates with the Patient-Reported Outcomes Measurement System Sleep Disturbance Bank (θ>0.85), which converges with the Pittsburgh Sleep Quality Index (r=0.85) [42]. They were also asked if they were concerned about their sleep (yes or no).

#### Statistical Analysis

Incomplete surveys or those with duplicate contact information were removed. Histograms of all variables were inspected. All drinking variables were negatively skewed with a high number of *0* cases, but repeating the analysis with these cases excluded did not alter the statistical outcomes.

Descriptive statistics (frequencies, means, and standard deviations) were calculated for all completed surveys and then separately for (1) ineligible web surveys, (2) eligible web surveys not enrolled in the study (phone screen failures or not interested), and (3) eligible surveys that were enrolled in the study. These 3 groups were then compared using the analysis of covariance for continuous variables (ie, age, sleep quality, AUDIT-C, heavy drinking episodes per 14 days, drinks per week) and chi-square for categorical variables (ie, social media platform referral source, each possible reason for interest, sex, brand of smartphone). Similar analyses of covariance were also performed comparing participants with respect to (1) referral from Snapchat versus the other platforms (to investigate sample features possibly contributing to the lower advertising efficiency of Snapchat that we observed) and (2) sex (to investigate sample features affected by the higher preponderance of women than men in our final sample). Analyses were conducted using a significance level of α<.05, and adjusted for age and sex where significant. We applied the Bonferroni correction to post hoc pairwise comparisons. The analysis of AUDIT-C data did not include the ineligible web survey group, because they were all below the high-risk cutoffs (≥5 women and ≥7 men), and the other groups were all at or above these cutoffs.

### Study 2: Comparing Effectiveness Between Advertising Themes

#### Advertising Experiment

**Figure 2:**
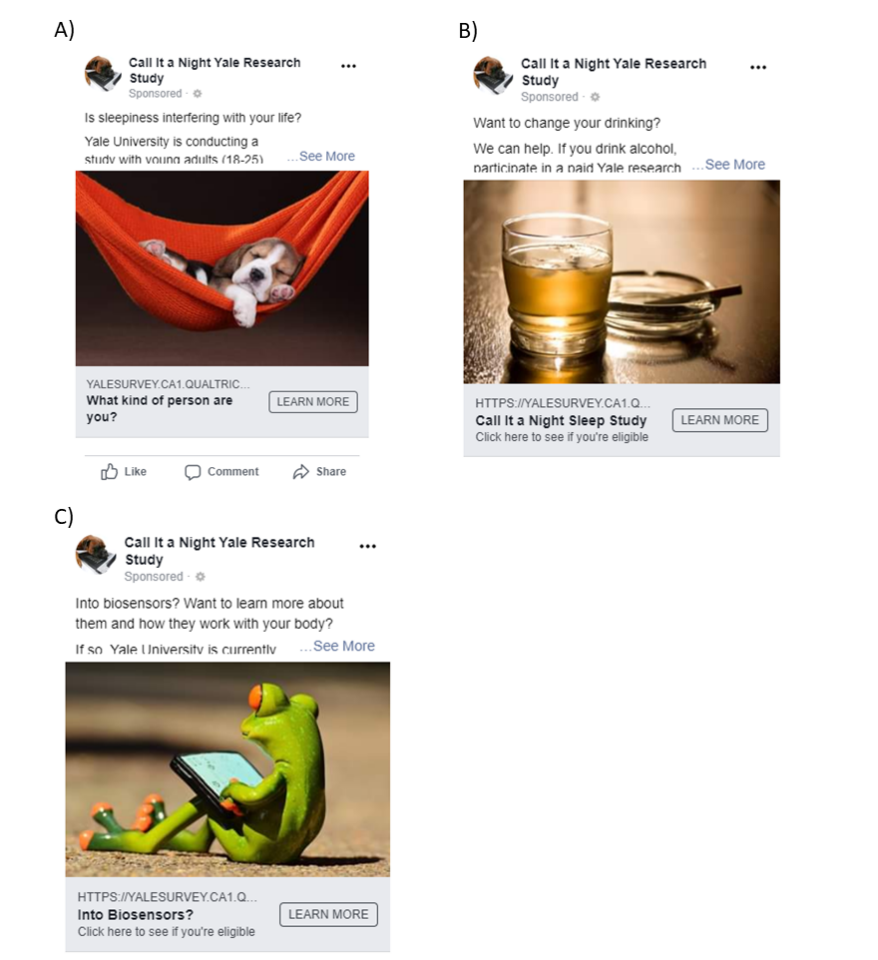
Examples from content-specific advertising sets to compare effectiveness between advertising themes (study #2). A) Sleep; B) Alcohol; C) Biosensor & health.

We generated one content-specific advertising set for each theme represented in the advertisements in study 1 (see the section *Facebook, Instagram, and Snapchat Recruitment* from study 1) so that we could isolate what was most effective (Figure 2): (1) *sleep* advertisements focused solely on sleep concerns without mentioning alcohol (eg, “Is sleepiness interfering with your life?”), (2) *alcohol* advertisements focused solely on alcohol concerns without mentioning sleep (eg, “Want to change your drinking?”), and (3) *biosensor and health* advertisements focused on the concept of multiple health behavior intersections without mentioning sleep or alcohol (eg, “Into biosensors? Want to learn more about them and how they work with your body?”). The *biosensor and health* set also served as a control arm accounting for attraction to health information, thus isolating the impact of attraction to the specific health topics of sleep and alcohol consumption. We uploaded them to Facebook/Instagram for 48 hours (October 5 and 6, 2019). Each advertisement set was displayed to users by Facebook’s auctioning and bid-optimizing process with an equal spending limit for each set (US $35 per day). Within each set, Facebook chose a specific advertisement for each impression by prioritizing those receiving the greatest number of clicks per impression (ie, a presentation-optimizing tool).

#### Statistical Analysis

To evaluate the relative success of different advertising content to attract interest, we compared the number of clicks generated per impression [43] among the 3 sets of advertisements tested for 2 days using a chi-square test with post hoc Bonferroni-adjusted pairwise comparisons.

## Results

### Study 1

#### Recruitment Results

**Figure 3:**
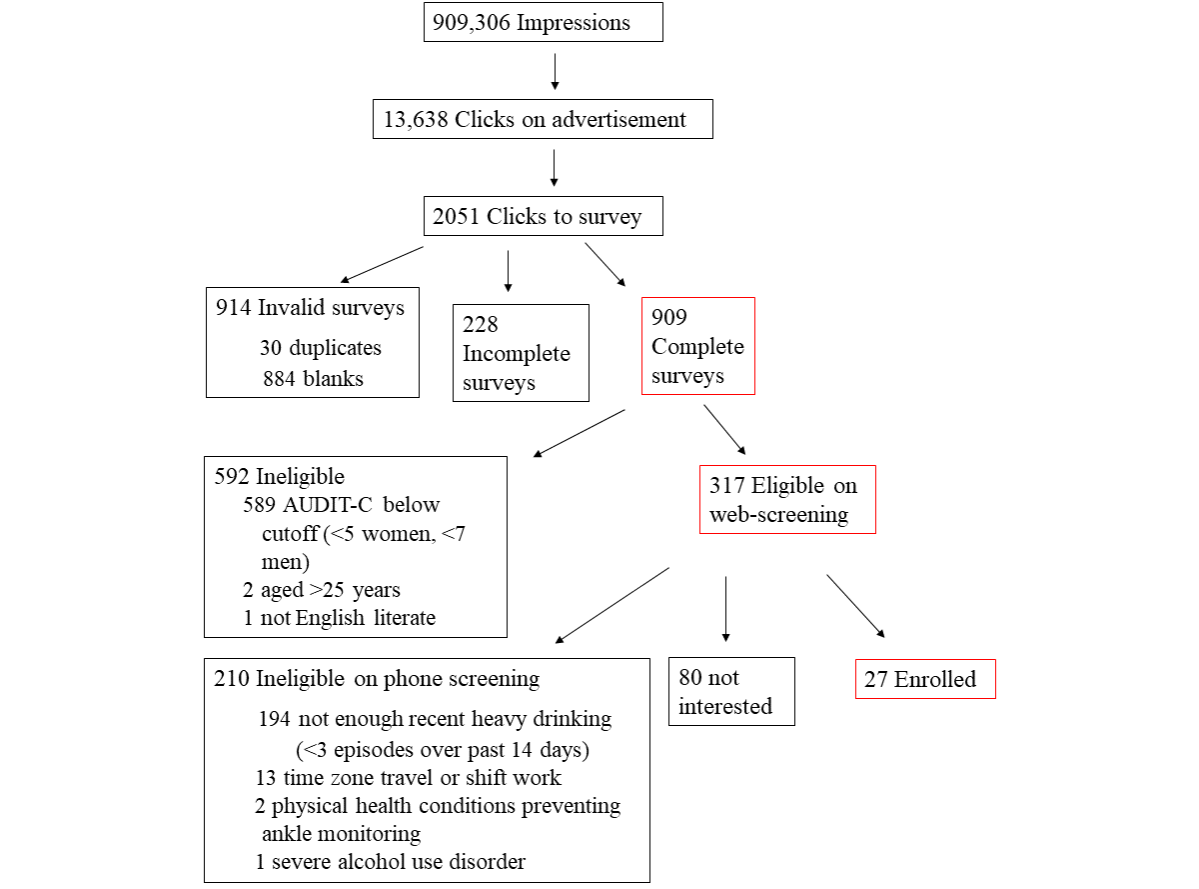
Flow diagram showing response rates to advertisements and outcomes of screening among responders.

The 4 Facebook/Instagram advertisements together were displayed 249,940 times (ie, impressions), clicked 4475 times, and yielded 1052 valid surveys in 3 months for a total cost of US $2013.28 (Figure 3). The number of unique users reached was 80,882 out of 220,000 Facebook/Instagram users in the set demographic (18-25 years of age in the New Haven area). One Snapchat advertisement, meanwhile, was displayed 659,366 times, swiped up 9126 times, and yielded 85 valid surveys in 3 months. The total cost was US $1610.70. The number of unique users reached was 140,142 out of 383,000 Snapchat users in the set demographic.

Facebook/Instagram advertisements were viewed mostly on mobile devices (4229/4475, 94.5% vs 215/4475, 4.8% on desktops and 27/4475, 0.6% on tablets), and Snapchat was delivered only on mobile devices. We removed blank (n=884) and duplicate contact information surveys (n=30), leaving 1137 valid entries for analysis. Among these, 228 (20.1%) answered initial questions about their source of referral and reason for interest in the study but did not complete demographic and drinking information. Compared with complete survey responders, these noncompleters expressed less interest (121/228, 53.1% vs 626/909, 68.9%) and concern about their sleep (142/228, 62.3% vs 725/909, 79.8%; *P*<.001) but similar interest (32/228, 14.0% vs 165/909, 18.2%; *P*=.14) and concern about alcohol (14/228, 6.1% vs 49/909, 5.4%; *P*=.66) and the connection between alcohol and sleep (135/228, 59.2% vs 547/909, 60.2%; *P*=.79). The source of study referral did not differ between survey completers and noncompleters (Facebook: 213/228, 93.4% vs 839/909, 92.3%; Snapchat: 15/228, 6.6% vs 70/909, 7.7%; *P*=.57).

Among the completed surveys (n=909), 317 volunteers met the preliminary drinking criteria (ie, AUDIT-C ≥5 women, ≥7 men) and were contacted by phone to verify their recent number of heavy drinking occasions (past 14 days) and final exclusion criteria. Many of these were excluded (n=210) or withdrew interest (n=80) during the screening process. The remaining 27 (ie, 8.5% of the 317 preliminarily eligible web screeners) attended an intake appointment and enrolled in the larger sleep intervention study.

Facebook/Instagram was more expensive per click than Snapchat (US $0.45 vs US $0.18), but less expensive per completed survey (US $2.40 vs US $23.01), initial positive eligibility screen (US $6.35 vs US $50.33), and enrollment (US $95.87 vs US $268.45). Thus, the average cost of enrolling 1 volunteer through the platforms combined was US $134.22. Some of these volunteers spread word-of-mouth referrals about the study, leading to the enrollment of 5 more volunteers (thus achieving the target of 32 volunteers) without further advertising. Counting this indirect return on investment, the average cost of enrolling 1 volunteer was US $113.25. The statistical comparisons reported below were unchanged with the 5 referred volunteers included.

Within the Facebook/Instagram platform, our advertisements were dramatically more successful on Facebook than on Instagram (US $0.43 vs US $0.79 per click) such that the bid-optimizing algorithm targeted 97.4% of the 249,940 impressions to the former.

#### Attraction Mechanisms and Referral Source

Most survey completers reported sleep concerns (725/909, 79.8%), and the majority reported interest in sleep (626/909, 68.9%) and in the connection between sleep and alcohol (547/909, 60.2%; Table 1). However, few reported interest in alcohol (165/909, 18.2%) and still fewer reported concerns about alcohol (49/909, 5.4%) or any other reason for interest in the study (30/909, 3.3%). Sleep concerns were equally prevalent among those who were ineligible, eligible upon web screening but not enrolled, and enrolled. The prevalence of alcohol concerns, however, was greater among those meeting the preliminary drinking criteria (ie, AUDIT-C scores) and still greater among the subset that met full eligibility criteria and enrolled.

An overwhelming majority of survey completers were referred by the Facebook/Instagram platform (839/909, 92.3%) rather than Snapchat (70/909, 7.7%; Table 1). However, this difference was attenuated among volunteers who enrolled (21/27, 78% Facebook/Instagram vs 6/27, 22% Snapchat). Within the Facebook/Instagram platform, 803/839 (95.7%) of referrals came from Facebook and only 4.3% (36/839) came from Instagram, consistent with the greater proportion of impressions targeted to Facebook (see the section Recruitment Results).

**Table 1:** Referral and attraction mechanisms.

Characteristics		Ineligible upon web screening	Eligible upon web screening but not enrolled	Enrolled	Chi-square *(df)*	*P* value
Participants, n		592	290	27	N/A^a^	N/A
**Referral source, n (%)**						
	Facebook or Instagram	554 (93.6)	264 (91.0)	21 (77.8)^b^	10.0 (2)	.01
	Snapchat	38 (6.4)	26 (9.0)	6 (22.2)	N/A	N/A
**Reason for interest, n (%)**						
	Sleep (interest)	438 (74.0)	174 (60.0)^b^	14 (59.1)^b^	21.5 (2)	<.001
	Sleep (concern)	466 (78.7)	235 (81.0)	24 (88.9)	2.1 (2)	.35
	Alcohol (interest)	73 (12.3)	83 (28.6)^b^	9 (33.3)^b^	39.1 (2)	<.001
	Alcohol (concern)	14 (2.4)	29 (10.0)^b^	6 (22.2)^b^	37.7 (2)	<.001
	Sleep-alcohol connection (interest)	294 (49.7)	232 (80.0)^b^	21 (77.8)^b^	78.4 (2)	<.001

^a^N/A: not applicable.

^b^Greater than ineligible upon web screening (Bonferonni-adjusted *Q*<0.05).

#### Demographic Characteristics, Sleep, and Drinking Characteristics

On average, those who completed the web-based survey were 21.1 (SD 2.3) years of age, and 69.4% (631/909) were female (Table 2). A substantial fraction (317/909, 34.9%) met the preliminary drinking criteria (ie, AUDIT-C score). Meeting the preliminary drinking criteria was associated with slightly worse subjective sleep quality. Those who met the preliminary drinking criteria but were later excluded or withdrew interest had lower AUDIT-C scores (*Q*<.001) and approximately 50% lower total drinks per week and frequency of heavy drinking than those who enrolled (*Q*=.01).

**Table 2:** Demographic, sleep, and drinking characteristics.

Characteristics		Ineligible upon web screening	Eligible upon web screening but not enrolled	Enrolled	Test statistic		*P* value
					*F* test (*df*)	Chi-square (*df*)	
Number of participants, n		592^a^	290	27	N/A^b^	N/A	N/A
Age (years), mean (SD)		21.1 (2.3)	21.2 (2.1)	20.3 (1.7)	2.352 (908)	N/A	.10
Sex (female), n (%)		392 (66.2)	223 (76.9)^c^	16 (59.3)^d^	N/A	11.8 (2)	.003
**Smartphone, n (%)**							
	iPhone	509 (86.0)	264 (91.0)	24 (88.9)	N/A	4.6 (2)	.10
	Android	83 (14.0)	26 (9.0)	3 (11.1)	N/A	N/A	N/A
Sleep quality on 1-5 scale, mean (SD)		2.8 (0.9)	2.7 (0.8)^c^	2.5 (0.9)	5.411 (827)	N/A	.005
AUDIT-C^e^ on 0-12 scale, mean (SD)		All below high-risk cutoffs (<5 women and <7 men)	6.6 (1.5)	8.0 (1.6)^d^	19.032 (316)^f^	N/A	<.001
Heavy drinking episodes in the past 14 days, mean (SD)		0.5 (0.8)	2.6 (2.3)^c^	5.1 (1.9)^c,d^	299.243 (908)^f,g^	N/A	<.001
Drinks per week, mean (SD)		3.9 (4.5)	13.7 (8.6)^c^	28.1 (27.4)^c,d^	288.710 (908)^f,g^	N/A	<.001

^a^81 volunteers from this group were excluded from the analysis of sleep quality because they chose not to answer this question. They were nonetheless counted as completed surveys because sleep quality did not affect study eligibility.

^b^N/A: not applicable.

^c^Different than ineligible upon web screening (Bonferroni-adjusted *Q*<.05).

^d^Different than eligible upon web screening but not enrolled (Bonferroni-adjusted *Q*<.05).

^e^AUDIT-C: consumption questions of the Alcohol Use Disorders Identification Test.

^f^Adjusted for sex.

^g^Adjusted for age.

#### Characteristics by Referral Source

Survey completers referred by Snapchat versus Facebook were younger and more likely to be male (Table 3). They were more likely to report sleep concerns and high-risk drinking. They had a tendency to report lower sleep quality, but it did not reach statistical significance.

**Table 3:** Participants compared by referral source.

Characteristics		Facebook	Snapchat	Test statistic			*P* value
				Chi-square (*df*)	*F* test (*df*)		
Participants, n		839	70	N/A^a^	N/A	N/A	
**Reason for interest, n (%)**							
	Sleep (interest)	582 (69.4)	44 (62.9)	1.3 (1)	N/A	.26	
	Sleep (concern)	662 (78.9)	63 (90.0)	4.9 (1)	N/A	.03	
	Alcohol (interest)	154 (18.4)	11 (15.7)	0.3 (1)	N/A	.58	
	Alcohol (concern)	44 (5.2)	5 (7.1)	0.5 (1)	N/A	.50	
	Sleep-alcohol connection (interest)	501 (59.7)	46 (65.7)	1.0 (1)	N/A	.33	
Age (years), mean (SD)		21.1 (2.2)	19.9 (2.2)	N/A	19.627 (908)	<.001	
Sex (female), n (%)		596 (71.0)	35 (50.0)	N/A	13.468 (1)	<.001	
**Smartphone, n (%)**							
	iPhone	732 (87.2)	65 (92.9)	1.9 (1)	N/A	.17	
	Android	107 (12.8)	5 (7.1)	N/A	N/A	N/A	
Sleep quality on 1-5 scale, mean (SD)^b^		2.8 (0.9)	2.6 (0.9)	N/A	3.714 (827)	.05	
AUDIT-C^c^ above high-risk cutoff (≥5 women and ≥7 men)		284 (33.9)	32 (45.7)^b^	4.0 (1)	N/A	.047	

^a^N/A: not applicable.

^b^Excludes 9% (76/839) of Facebook volunteers and 7% (5/70) of Snapchat volunteers because they chose not to answer this question.

^c^AUDIT-C: consumption questions of the Alcohol Use Disorders Identification Test.

#### Characteristics by Sex

Compared with men, women had a less prevalent concern and interest in alcohol, but a greater prevalence of heavy drinking (Table 4). They were also more likely than men to use iPhones versus Android smartphones.

**Table 4:** Participants compared by sex.

Characteristics		Women	Men	Test statistic		*P* value
				Chi-square (*df*)	*t* test (*df*)	
Number of participants		631	278	N/A^a^	N/A	N/A
**Reason for interest, n (%)**						
	Sleep (interest)	433 (68.6)	193 (69.4)	0.1 (1)	N/A	.81
	Sleep (concern)	497 (78.8)	228 (82.0)	1.3 (1)	N/A	.26
	Alcohol (interest)	101 (16.0)	64 (23.0)	6.4 (1)	N/A	.01
	Alcohol (concern)	26 (4.1)	23 (8.3)	6.5 (1)	N/A	.01
	Sleep-alcohol connection (interest)	368 (58.3)	179 (64.4)	3.0 (1)	N/A	.09
Age (years), mean (SD)		21.1 (2.3)	21.0 (2.2)	N/A	1.422 (908)	.23
**Smartphone, n (%)**						
	iPhone	572 (90.6)	225 (80.9)	16.9 (2)	N/A	<.001
	Android	59 (9.4)	53 (19.1)	N/A	N/A	N/A
Sleep quality on 1-5 scale, mean (SD)^b^		2.8 (0.9)	2.7 (0.9)	N/A	1.011 (827)	.315
AUDIT-C^c^ above high-risk cutoff (≥5 women and ≥7 men)		238 (37.8)	78 (28.2)	7.9 (1)	N/A	.005

^a^N/A: not applicable.

^a^Excludes 9% (56/631) of women and 9% (25/278) of men because they chose not to answer this question.

^b^AUDIT-C: consumption questions of the Alcohol Use Disorders Identification Test.

### Study 2

Over the 2 days that we ran content-specific advertising sets with the same platform settings and spending limits, each set received a similar number of impressions, indicating similar efficiency of dissemination (*sleep* 12,523; *alcohol* 11,629; *biosensor and health* 12,825). However, the advertisement set *sleep* generated more clicks per impression (114/12,523, 0.91%) than the other sets (*alcohol* 65/11,629, 0.56%; *biosensor and health* 64/12,825, 0.51%; *Q*<.001).

## Discussion

### Principal Findings

The results of this study provide overall evidence for the effectiveness of social media advertising for recruiting heavy-drinking young adults to engage in treatment and specifically for content focused on sleep. Poor sleep quality and heavy drinking behaviors both occurred for a substantial fraction of the sample. However, sleep concerns were significantly more common than drinking concerns and appeared to drive engagement in the web screening process. In particular, the sleep advertisement performed better than the drinking advertisement as an advertising hook to generate initial clicks, and sleep, but not drinking concerns was associated with completion of the survey beyond the first two pages. Among the heavy drinkers identified by the web screener, more than one-third passed the further eligibility criteria, and there was strong enrollment uptake among this finally eligible group. Many heavy-drinking young adults report being unconcerned about their drinking [7,8], but our findings indicate that this cohort may nonetheless be concerned about their sleep, which could be a novel on-ramp to engage them in drinking-related treatment. The most common reason for failing to meet the eligibility criteria after the web screening process was acute recent drinking patterns that were too low to meet the study criteria (<3 heavy episodes in the past 14 days) despite chronic drinking patterns that were high risk according to the AUDIT-C scores. The main study required ≥3 heavy drinking episodes in the past 14 days to test the effect of a mobile sleep intervention for the greater at-risk population of young adult heavy drinkers. Using this broader criterion (ie, AUDIT-C scores) would approximately triple the number of eligible volunteers that we could have enrolled.

### Comparison With Previous Work

Previous research has demonstrated the cost-effectiveness of Facebook recruitment. A previous systematic review of 27 studies utilizing Facebook recruitment reported that the median cost of enrolling an eligible candidate was US $14.41 [43], which is substantially cheaper than our findings. The possible factors that can elevate cost are low engagement (clicks per impression), conversion (surveys completed per click), eligibility (surveys eligible per surveys completed), and enrollment (volunteers enrolled per eligible volunteers). Our rate of engagement outscored 57% (13/23) of the studies reviewed (ie, our cost per click was US $0.45 vs a median of US $0.51), and our rate of conversion outscored 90% (18/20) of the studies reviewed (ie, our cost per completed survey was US $2.40 vs a median of US $12.00). However, our rate of eligibility was lower than that of every study reviewed (ie, 13% vs a median of 61%). Once eligible candidates were identified, our rate of enrollment was strong for a behavioral intervention clinical trial (25% (27/107) of eligible volunteers enrolled). In summary, the driver of our high cost was the low proportion of survey respondents who met the eligibility criteria. However, as noted above, this would have tripled if we only screened for AUDIT-C scores without requiring a high concentration of heavy drinking in the past 14 days. In that instance, our cost per enrolled volunteer would have been approximately US $32.

Other important factors could have accounted for the cost-effectiveness of the results. For instance, our main study involved participation in an in-person intervention requiring a total of six contacts. In comparison, most previous studies required less volunteer commitment; most involved brief web-based assessments or interventions. These factors could have attenuated the engagement, conversion, and enrollment of our recruitment process and driven up costs.

Costs were higher for Snapchat than for Facebook, as also seen in the only previous study comparing the two platforms for recruitment of youth for research [21]. However, the reasons for this greater cost remain unclear. Snapchat is gaining popularity among young people relative to Facebook [30] and has the potential to be a valuable recruitment tool. Snapchat users were not inferior study candidates than Facebook users. In fact, survey completers referred by Snapchat versus Facebook were significantly more likely to report sleep concerns and high-risk drinking and had a nonsignificant tendency to report lower sleep quality (Table 3). One could speculate that Snapchat’s overall greater uses and gratifications around personal disclosure [32-34] facilitated this personal disclosure about health behaviors and a subsequent interest in treating them through our study. Demographic differences also do not explain Snapchat’s higher cost: survey completers referred by Snapchat versus Facebook were significantly younger and more likely to be male (Table 3), characteristics that were associated with a tendency for greater enrollment in our sample (Table 2). Differences in geographic radius are also unlikely to explain Snapchat’s higher cost: Snapchat advertisements did not need to be extended as far as Facebook advertisements to attain a meaningful number of impressions (10 miles vs 25 miles), indicating that Snapchat users were in closer proximity to New Haven, thus even better positioned to attend the office visits.

Therefore, a more likely explanation for Snapchat’s greater cost may be logistical differences between the platforms that we lacked resources to experimentally control given that directly comparing Facebook and Snapchat was not the primary aim of our study. The first such difference was that during analysis 1 (ie, January-April, 2019), Facebook was more restrictive than Snapchat regarding alcohol-related advertising content for users aged <21 years. Our Facebook advertisements during this window placed greater relative emphasis on sleep than alcohol, whereas the Snapchat advertisement had a more balanced emphasis between the two (Figure 1). This difference could have been a confounder, as study 2 later revealed the superiority of sleep content in attracting clicks. The second logistical difference between Facebook and Snapchat was that the latter required a greater number of informational landing pages separating the advertisement from the survey questions. This requirement, imposed because Snapchat found our privacy policy lacked enough explanation to be directly linked from an advertisement, could have deterred survey completion. On the positive side, it filtered some users not engaged enough to follow through with enrollment, evidenced by the lesser cost difference when tabulating enrolled volunteers as opposed to completed surveys. However, this only partially rebalanced the cost difference between the platforms. Future studies could elicit specialized customer support from Facebook and Snapchat to control these logistical differences, thus making a more valid direct comparison. As for Instagram, it was outperformed by Facebook advertisement placement, but the shared platform bid-optimizing algorithm automatically solved this problem by targeting advertisement impressions away from Instagram to Facebook. Although image-based social media, such as Instagram, previously led to decreased loneliness and increased happiness and satisfaction with life compared with Facebook among young adults [31], we found it was less effective as a recruitment tool.

### Limitations

This study had several limitations. First, the in-person nature of the study limited geographic representation. Second, social media advertising cannot ensure representative sampling because click rates are low. Furthermore, we acknowledge that these low click rates could reflect this being a hard-to-reach population that may be better reached by online community-based or respondent-driven sampling [44]. On the other hand, our click rates were above the median of previous studies that included easier-to-reach populations [43], suggesting this was also an appropriate population for which to explore social media advertising. In addition, we successfully oversampled heavy drinkers compared with survey data [45]. Third, alcohol use risk status was limited to self-report. Fourth, we studied predictors of advertisement clicking but not survey completion (eg, survey user experience). Fifth, a disproportionate number of respondents were women (69%). However, the women in our sample had significantly more prevalent heavy drinking and less prevalent concern about drinking than men (Table 4), thus their greater inclusion may be a natural consequence of our objective to target high-risk drinkers who are not concerned about their drinking. Sixth, although we designed advertising content to achieve the aims of both studies, style is a possible confounder. In study 1, although the text mentioned both sleep and alcohol, the visuals were more focused on sleep, which could have biased attention toward survey responses reflecting greater sleep concerns and lesser alcohol concerns. However, before accessing the survey, participants had to view several layers of text (advertising text and study overview text on the following page), which extensively referenced both sleep and alcohol as integral to the inclusion criteria and intervention. Thus, survey respondents had been exposed to information on both sleep and alcohol. We therefore maintain that our analysis demonstrates greater concerns about sleep than alcohol. In study 2, the sets had stylistic differences (visual placement, size of images, and wording) that could have confounded the content differences that we aimed to examine. However, the 27 enrolled participants were queried on poststudy interviews as to what they found appealing about the advertisements, and none mentioned image or stylistic aspects (full content of interviews to be published in a forthcoming manuscript). The final limitation was that we reached more college students than nonstudents.

### Conclusions

Despite these limitations, these data demonstrate that social media advertisements targeting young adults with sleep concerns or an interest in sleep reach those who drink heavily and are more effective than advertisements focused on drinking. Many young adults do not seek help for their drinking because of several potential reasons, including low perceived need [46]. Thus, having another on-ramp for alcohol prevention strategies is important. In this case, targeting a coexisting health behavior that young adults may be more open to discuss (ie, sleep) may facilitate better engagement regarding their drinking, a behavior they may be open to discussing. We previously found that heavy-drinking young adults find sleep interventions appealing, are interested in personalized information about sleep and alcohol interactions [15], and that sleep interventions demonstrate promise as a gateway for intervening in alcohol use and engaging heavy-drinking young adults in treatment [16]. Data from this study lend insight into the scalability of this approach by demonstrating that it can be disseminated using social media. We could further infer from this study that a sleep intervention could be disseminated using other web-based venues that are becoming widely popular among young adults (eg, web-based information, mobile apps, and support groups [18]). In addition, among young adults, sleep and alcohol have been found to cluster not only with each other but also with other health behaviors such as smoking and diet. Furthermore, interventions changing one of these behaviors sometimes change others as well [47]. Thus, the proof-of-concept generated by our past work and this study bears potential for extension to these other behaviors. In addition, this line of research warrants further study across other popular social media platforms, such as YouTube and Twitter [48].

## Abbreviations

AUD: alcohol use disorder
AUDIT-C: consumption questions of the Alcohol Use Disorders Identification Test
